# Vaccine research and development capacity in Central and West Asia: A path toward sustainable vaccine R&D programs

**DOI:** 10.3389/fpubh.2023.1143790

**Published:** 2023-03-02

**Authors:** Jonathan Hare, Giovanna Riggall, Alexander Bongers, Kirthi Ramesh, Larissa Kokareva, Brian Chin

**Affiliations:** ^1^Crown Agents, London, United Kingdom; ^2^Asian Development Bank, Manila, Philippines

**Keywords:** vaccines, research, LMIC, capacity, development, Central Asia, West Asia

## Abstract

The ability to support a comprehensive vaccine research and development (R&D) portfolio from a health security perspective has taken on enhanced significance over the past 3 years whereby countries that had existing vaccine R&D infrastructure (G7, Russia and China) have been at the forefront of global efforts to combat COVID-19. Few countries outside of these key players have the infrastructure necessary to develop national vaccine programs, though this is beginning to change with investment across many low- and middle-income countries. These same opportunities exist for countries in Central and West Asia, and in this perspective, we highlight the existing infrastructure and expertise across seven countries (Armenia, Azerbaijan, Georgia, Kazakhstan, Kyrgyzstan, Tajikistan, and Uzbekistan) and propose opportunities for enhanced collaboration along with a bold proposal for establishing a new-build, regional vaccine translational research institute to facilitate the development of a robust, regional vaccine R&D environment to combat existing and future health challenges.

## 1. Introduction

Vaccine research and development has experienced a revolution following the emergence of the SARS-CoV-2 as the causative agent of coronavirus disease 2019 (COVID-19), with previously unprecedented multinational efforts to develop and deploy safe and effective vaccines globally (1). Three years after the initial outbreak of COVID-19 first identified in Wuhan, China (2), this effort has translated into massive global vaccination campaigns which has contributed to the reversal of the pandemic curve in many countries although the health and financial impacts of the virus remain enormous. COVID-19 has caused over 6.6 million reported deaths to date (3). There has also been a colossal economic impact of the pandemic, with an estimated 6.5% drop in gross domestic product globally in 2020 and a projected total economic loss worldwide of US$ 28 trillion by 2025 (4).

The impact of COVID-19 has been exaggerated by the significant inequalities in access to vaccination and health and research infrastructures, accentuating the gap between developed and low- and middle-income countries (LMIC). This discrepancy is across all vaccines with the latest research from WHO indicating that only 41% of low-income countries have received the human papilloma virus (HPV) vaccine compared to >80% for high income countries, despite a comparable disease burden (5). These inequalities, in addition to posing a serious threat to the management of current and future infectious disease risks, including the ongoing pandemic (6) have had particular impact in children and young people (CYP) where, notwithstanding the low uptake of childhood COVID-19 vaccination in LMIC, it is currently estimated that during the pandemic ~8 million CYP have been deprived of life-saving, routine childhood immunizations (7).

Addressing these inequalities will require leadership at a global, regional, and country level and whilst there are still limited examples of successful in-country programs, Russia's invasion of Ukraine has accelerated interest in investigating whether the Central and West Asia region can support more vaccine research (8). In 2021, the Vaccine Advisory Firm for Central and West Asia project was established by the Asian Development Bank and implemented by Crown Agents in partnership with FHI 360. The project provides technical assistance and advisory support to Armenia, Azerbaijan, Georgia, Kazakhstan, Kyrgyzstan, Tajikistan, and Uzbekistan (9). In this perspective, we review the status of vaccine research and development in these seven countries in Central and West Asia and propose a novel solution for consideration in developing future vaccine R&D resilience and move to a sustainable model of vaccine independence.

## 2. Vaccine R&D capacity: Country and regional analysis

In-country vaccine development capabilities were modest in all the countries evaluated. Kazakhstan had the most advanced programs in both clinical development and vaccine manufacturing capabilities with published examples of vaccine development humans (10) and veterinary settings (11). Further examples of vaccine research and clinical development exist in Azerbaijan (reviewed further Section 2.1) and Uzbekistan (12) where the COVID-19 vaccine ZF-UZ-VAC 2001 was developed in collaboration with the Chinese company Anhui Zhifei Longcom Biopharmaceutical (13). Limited information exists on the status of vaccine R&D in Kyrgyzstan, with available published data consisting of collaborations on veterinary with groups in Kazakhstan (14). Direct outreach to Republican Center for Immunoprophylaxis indicated that there are no ongoing vaccine R&D activities within Kyrgyzstan. There is no available evidence of in-country programs in Armenia, Georgia, Tajikistan, and Turkmenistan.

The distribution of COVID-19 vaccine provision with each of the seven participating countries is described in Table 1. The column bilateral/multilateral agreements include vaccines produced through any national vaccine R&D programs in addition to agreements with external vendors and/or countries and, by extrapolating the numbers of vaccines procured from documented, regional provided can be implemented as a crude surrogate for internal vaccine R&D capabilities. As a percentage of the total vaccine received in each country, this figure ranges from 0 to 100%. This range adjusts to a maximum of 4% if Uzbekistan is excluded. Vaccine doses produced in country account for ~48% of all doses administered in Uzbekistan (15). The procurement and deployment of COVID-19 vaccines shows improvement on the previous Human Papilloma Virus (HPV) vaccination campaigns whereby vaccine availability was limited to Armenia, Georgia, and Uzbekistan (16) with vaccines sourced primarily through GAVI from multinational pharma (17) and indicates that there may be a nascent vaccine infrastructure that could be leveraged to improve capacity throughout the region.

**Table 1:** Source of COVID-19 vaccines administered in 7 countries in Central and West Asia.

**Country**	**Bilateral/multilateral agreements**	**Donations**	**COVAX**	**Unknown**	**Total**
Armenia	0.77 (0.08)	1.62	0.38	0.18	2.97
Azerbaijan	0.43 (0.08)	0.15	2.02	12.64	15.24
Georgia	2.62 (0)	0.80	0.16	0	3.59
Kazakhstan	7.54 (0.02)	0.15	0	15.31	23.01
Kyrgyzstan	2.47 (0.12)	2.27	1.33	0	6.07
Tajikistan	0	0.54	12.58	4.46	17.58
Uzbekistan	39.15 (39.15)	0.65	25.45	16.09	81.35

Numbers are in millions of doses. Values in parentheses are doses of either Sputnik V or doses sourced from Anhui Zhifei. Source data: UNICEF COVID-19 Market Dashboard, correct as of 14 December 2022 (15).

To further quantify the available vaccine R&D capabilities in each country, in addition to the open access literature search, detailed outreach was initiated to all participating countries to further understand their capabilities. Responses to this outreach was received from three participating countries: Azerbaijan, Georgia, and Tajikistan.

### 2.1. Country specific analysis: Azerbaijan

Evaluation of the vaccine R&D capabilities, subsequently confirmed by the Azerbaijan MOH, revealed that, at a government level, Azerbaijan has not actively been involved in the production of vaccines since the country's independence from the former Union of Soviet Socialist Republics (USSR) in 1991. A literature and landscape review using publicly available information focused on vaccine development identified five peer-reviewed publications with Azerbaijan-based authors at either university, research institutes or in named biotech companies in the period 2016–2021. Ongoing vaccine R&D activities are focused around designing potential cancer vaccines (18), evaluating adjuvants for poultry vaccines (19) and development and testing of a malaria vaccine to Pfs48/45 (20). Further vaccine R&D activities indicate development of pharmaceuticals using plant transient expression technology (21) with several vaccine candidates developed to COVID-19 (22) and therapeutic candidates such as human furin factor IX (23).

### 2.2. Country specific analysis: Georgia

A literature and landscape review revealed no peer-reviewed publications with Georgian-based authors at either university, research institutes or in named biotech companies in the period 2016–2021 focused on vaccine development. Through research conducted by local representatives, two institutions were identified that may have the capacity and expertise to support local and regional vaccine R&D activities. These institutions are the Richard Lugar Center for Public Health Research, within the National Center for Disease Control and Public Health, an institution focused on researching human and animal pathogens and the George Eliava Institute (GEI), a research institute based in Tbilisi that has historically focused on bacteriophage research. Combined, these institutions have existing facilities that could be leveraged to reengage vaccine R&D at a country level and as part of a wider regional collaboration.

### 2.3. Country specific analysis: Tajikistan

A literature and landscape review using publicly available information on vaccine development revealed no peer-reviewed publications with authors based at either universities, research institutes or in named biotech companies in Tajikistan in the period 2016–2021 on vaccine development. The Tajik National University (TNU) hosts the Biotechnology Center which focuses primarily on combating bacterial and viral diseases of agricultural crops. Contact was initiated with representatives within the Institute of Biological Safety Problems and Biotechnology (IBSPB) and the Biotechnology Center (BC) under the TNU to better understand the available capabilities. The outcome of this research is that, whilst there are some available infrastructures, there is an expertise deficit that would need to be remedied prior to considering further innovation. To further examine capabilities at selected institutions, a questionnaire was provided to all the contacts to evaluate the capabilities and expertise at each site.

### 2.4. Detail regional analysis

The questionnaire provided was broken down into three sections: (i) Section 1 covered the institutional expertise available at each of the participating institutions with respect to pathogen research, vaccine R&D and operational capabilities; (ii) Section 2 covered the institutional infrastructure available at each of the participating institutions that responded to this section, including laboratory capabilities, operational systems, and resourcing; and (iii) Section 3 explored in more detail the facilities available at each of the participating institutions that responded to this section, primarily laboratory equipment. Respondents were asked to complete all sections to the best of their knowledge.

**Figure 1:**
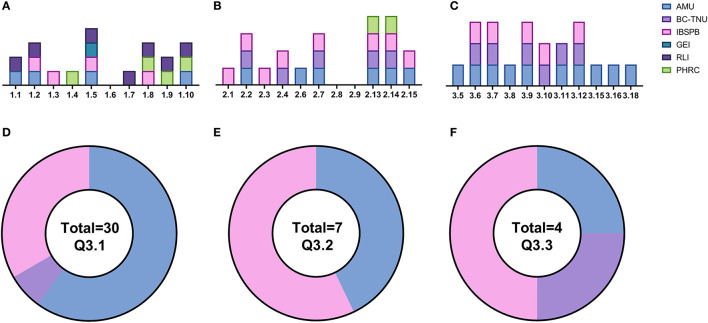
Summary of questionnaire responses. **(A)** Section 1: institutional/organizational expertise. **(B)** Section 2: laboratory infrastructure and personnel. **(C)** Section 3: technical expertise. **(D)** Distribution of fridges. **(E)** Distribution of −20°C freezers. **(F)** Distribution of −80°C freezers. AMU (blue): Azerbaijan Medical University (Azerbaijan), BC-TNU (mauve): Biotechnology Center-Tajik National University (Tajikistan), IBSPB (pink) = Institute of Biological Safety Problems and Biotechnology (Tajikistan), GEI (teal): George Eliava Institute (Georgia), RLI (purple): Richard Lugar Institute (Georgia), PHRC (green): Public Health and Reform Center (Azerbaijan).

Figure 1 describes the results of the survey and breaks down the responses by institution and questionnaire section. Azerbaijan Medical University (AMU) reported the most diverse experience in vaccine R&D, with confirmed expertise present in human immunobiology, pathogen biology and antibody discovery. These responses were broken down further to reveal broad expertise in T-cell, B-cell, and innate cell biology along with expertise in both bacteria and virus biology as well as antibody screening. The Institute of Biological Safety Problems and Biotechnology (IBSPB) of Tajikistan indicated they have experience in different vaccine technologies including adenovirus, DNA and RNA vaccine vectors as well as functional antibody assays. Whilst there is operational expertise in pre-clinical study design, Good Practice (GxP) regulatory experience, document management systems and quality assurance distributed throughout the different organizations, an infrastructure gap was identified relating to expertise in performing pre-clinical studies. The assessment of the specific equipment available at each of the four responding institutions indicates that only one institution (IBSPB) has experience in participating with vaccine R&D and has facilities that reflect this. AMU has comparable facilities, but no Biosafety Level 3 (BSL3) labs, and they are the only institute with expertise in an operational Laboratory Information Management System (LIMS). All the institutions report issues in obtaining consumables which makes expanding and developing vaccine R&D programs within individual countries a challenge and is something that needs to be considered vs. a regional approach where centralized and bulk ordering can be developed. Additionally, all the institutions reported challenges in cold chain, particularly around the availability of a consistent liquid nitrogen source.

## 3. Recommendations

### 3.1. Azerbaijan

The equipment at AMU would need significant upgrade to expand vaccine R&D capabilities. The absence of specialist protein analysis equipment, as well as molecular biology techniques and multiplexing technologies is a significant barrier to developing in-country vaccine R&D programs. Furthermore, the same infrastructure would have to be deployed at institutions within the contributing countries to improve in-country expertise.

The institutions in Azerbaijan had the most advanced infrastructure with laboratories at AMU providing most of the available equipment and facilities. In assessing the available resource, significant gaps remain that would need to be resolved if there is a desire to pursue innovative vaccine R&D programs within Azerbaijan. Further interrogation of the capabilities of the available equipment for performing cellular phenotyping would need to be clarified to see if additional equipment in addition to obtaining equipment for molecular and protein characterization is necessary. A detailed evaluation of the available floor plans would be necessary for developing accurate assumptions as to possible budgets required, but based in available literature, a conservative estimate would be for infrastructure investment more than US$2 million (24). In addition, there would also need to be a program of expanding expertise, which in the short term at least would likely have to be met through expatriate appointments.

Regional sharing of equipment and facilities through formal partnerships would be something that could add immediate additional capabilities. Developing agreements to access the BSL3 labs in either Georgia or Tajikistan would add immediate functionality to any R&D programs. However, a detailed feasibility study would be needed to determine whether additional infrastructure investments are needed to support any collaborative arrangements including additional staff training and assessing available logistics and clarifying customs requirements.

### 3.2. Georgia

As no formal responses to the full questionnaire were received from either the George Eliava Institute or the Richard Lugar Institute and therefore it was not possible to provide any specific recommendations should these institutes wish to expand their vaccine R&D activities. However, exploring whether formal partnerships could be established with both Azerbaijan Medical University (AMU) and the Institute of Biological Safety Problems and Biotechnology (IBSPB) of Tajikistan would have tangible benefits for future research programs.

### 3.3. Tajikistan

The institution with the most advanced infrastructure for vaccine R&D capabilities was the Institute of Biological Safety Problems and Biotechnology (IBSPB) of Tajikistan, however the facilities available are at the more basic end. Significant investment would be required to upgrade the facilities and infrastructure to a level where independent research programs could be sustained. Without a more detailed understating of the available floor plans it is difficult to make accurate assumptions as to possible budgets required but a conservative estimate for infrastructure investment would be over US$3 million. Any reciprocal arrangement that could be developed with institutions in the region, notably Azerbaijan Medical University (AMU), could offer a short-term mechanism to develop vaccine R&D programs. However, there are limitations to this collaborative approach, and they cannot substitute for infrastructure investment both nationally and regionally.

In addition to the country analysis described, outreach was attempted to the other participating countries, but we were not able to reach agreement with the relevant institutions. Information on the available expertise and capabilities within the region would add further to the case for a regional approach on vaccine R&D to combat existing and future health challenges in the region. One opportunity to further assess potential in the region is to leverage the Central Asia Regional Economic Cooperation (CAREC) platform that supports deepening regional collaboration in health including enhancing access to medical goods under its Health Strategy (25, 26). Six out of the seven countries covered in this paper are currently members of this platform.

### 3.4. Regional approach: A new-build, vaccine translational research institute

Vaccine development costs remain static whether implemented at an individual (company, university) or a country level (24, 27). Compared to developing country specific programs, a more cost-effective model for advancing a vaccine R&D program would be to share the costs of vaccine and therapeutics development between participating countries. This approach is currently being implemented in West Africa where the US Development Finance Corporation (DFC) is providing a US$ 3.3 million technical assistance grant to Fondation Institut Pasteur de Dakar (IPD) to support development of a vaccine production hub that will serve Senegal and the other countries of West Africa and aligns with a recently proposed solution for addressing vaccine inequities around the world (28).

In assessing the capacity for vaccine R&D within the participating countries revealed a nascent industry existing within an environment in need of significant infrastructure investment for it to develop further. The investment required for advancing vaccine R&D is considerable and whilst some countries may be further advanced, particularly Uzbekistan, many countries would have to invest significantly if they wanted to develop their national infrastructure with limited opportunities for leveraging cross-country cooperation. To try and leverage resources, we recommend the establishment of a new-build regional translational vaccine research institute that would look to translate ongoing research activities in the space of vaccine R&D at universities and institutes within participating countries in the Central and West Asia region (and potentially beyond) and develop them through to a clinical grade product that could be evaluated in Phase I/Phase II clinical trials within a 5-year time frame. Establishing such an institute may also bring tangential benefits including providing a range of job opportunities across multiple sectors as well as serving as a catalyst for future investment in social infrastructure and healthcare (29). By employing a regional cooperative strategy, we assess that a minimum investment of ~US$ 300 million over a 6-year period would be necessary for the development and implementation of this strategy. This figure compares favorably against recently announced similar initiatives including the US$ 2 billion award to the development of the Strategic Center of Biomedical Advanced Vaccine Research and Development for Preparedness and Response (SCARDA) in Japan (30) and the CAN$ 1.2 billion committed by the government of Canada for developing biomanufacturing and life sciences sector to prepare for future pandemics (31).

This idea of developing a new-build, vaccine translational research institute within Central and West Asia is ambitious and would require significant will at a political level and investment to achieve, but the benefits could be transformational for a region where vaccine R&D lags significantly behind comparable countries in other parts of the world and could contribute to resilience against future global vaccine shortages and a sustainable model of vaccine independence. Initial steps toward implementation would be to commission a feasibility study under the framework of CAREC health cooperation on a regional approach to vaccine R&D programs and the potential of developing a new-build, vaccine translational research institute.

## Data availability statement

The raw data supporting the conclusions of this article will be made available by the authors, without undue reservation.

## Author contributions

GR, LK, JH, AB, and BC conceptualized the study. JH reviewed the literature, conducted the research and analysis, wrote the initial draft of the manuscript, and made subsequent edits. BC, GR, AB, KR, and LK reviewed and edited the manuscript. All authors read and approved the final manuscript.
